# Workplace Culture and Biomarkers of Health Risk

**DOI:** 10.3390/ijerph191911920

**Published:** 2022-09-21

**Authors:** Brad Shuck, Joy L. Hart, Kandi L. Walker, Jayesh Rai, Shweta Srivastava, Sanjay Srivastava, Shesh Rai, Aruni Bhatnagar, Rachel J. Keith

**Affiliations:** 1 College of Education and Human Development, University of Louisville, Suite #346, Louisville, KY 40292, USA; 2 Department of Communication, College of Arts and Sciences, University of Louisville, Louisville, KY 40292, USA; 3 Christina Lee Brown Envirome Institute, University of Louisville, Louisville, KY 40202, USA; 4 Division of Environmental Medicine, School of Medicine, Louisville, KY 40202, USA; 5 Brown Cancer Center, Biostatistics and Bioinformatics Facility, University of Louisville, Louisville, KY 40202, USA; 6 Biostatistics and Informatics Core, Center for Integrative Environmental Health Sciences, University of Louisville, Louisville, KY 40202, USA; 7 Department of Bioinformatics and Biostatistics, University of Louisville, Louisville, KY 40202, USA

**Keywords:** work determinants of health, work culture, work environment, health risk, chronic disease, catecholamines

## Abstract

Workplace culture has been studied for impact on health risk; however, connections with robust biologic markers of health remain to be established. We examined associations between the work environment and urinary levels of catecholamines and their metabolites as biomarkers of sympathetic nervous system activity, indicative of stress. We recruited participants (n = 219; 2018–2019) from a cardiovascular risk cohort to investigate workplace culture, well-being, and stress. Participants completed seven questionnaires. Urine samples were used to measure catecholamines and their metabolites by LC/MS/MS. Pearson correlation and linear regression models were used after adjusting for demographics and creatinine. Participants reporting higher well-being had lower urinary levels of dopamine, serotonin, and 3-methoxytyramine. Participants reporting a more engaged and more positive workplace had lower levels of dopamine and 3-methoxytyramine. Reported workplace isolation was correlated with higher levels of dopamine and 3-methoxytyramine. Given correlations between catecholamines, we used 3-methoxytyramine for linear regression. In fully adjusted models, in environments with a more positive culture, levels of 3-methoxytyramine remained lower (β = −0.065 ± 0.025, *p* = 0.01) and indicated a positive association between workplace isolation and 3-methoxytyramine (β = 0.064 ± 0.030, *p* = 0.04). These findings are consistent with an important relationship between workplace environment and sympathetic nervous system activity.

## 1. Introduction

The workplace environment includes the natural environment, which encompasses location, environmental and pollution exposures; the social environment, which encompasses interactions, overall and sub-cultures, and organizational climate; and the personal environment, which encompasses personal relationships and individual choices [1]. One aspect of the overall workplace environment is workplace culture, which involves the experiences, values, and behaviors that contribute to the unique socio-psychological environment of an organization. Workplace culture has the potential to affect much of the US population. In 2020 most US families had one employed member and nearly 50% had two employed members [2], with work being the single largest source of time spent outside the home. Although national policy acknowledges the influence of work on a person’s overall health and health risk [3,4,5], notably missing are assessments of the potential for workplace culture to impact employee long-term health outcomes. Therefore, inquiry into *Work Determinants of Health*™ [6], or more specifically factors beyond traditionally explored occupational exposures, might better account for generalized vulnerability to disease and disparities in health risk.

Workplace health frequently has seen larger employers offering thematic programming for wellness with the end goal of preventing disease and lowering health-related costs, such as insurance and absenteeism [7]. Such programming often is geared toward traditional disease risk modifiers, is designed to be undertaken by employees, such as healthy living, diet, stress reduction, gym memberships, or onsite facilities, and fails to meet expectations or show positive health returns [8]. Few such programs address the underlying environment—or culture—within the workplace. Wellness programs are not enough to build environments for health, and many fail to address the root causes of work-based risks associated with the workplace environment, such as workplace culture. Our work focuses on the social and organizational aspects of the workplace environment, a feature traditional wellness programs often ignore. To be comprehensive in exploring the social and organizational aspects of the workplace environment, we operationalize and examine workplace culture by assessing four unique experiences to include: (a) how connected or (b) isolated a person feels in their workplace, (c) how engaged they were, and (d) conditions of work culture and the overall environment. These aspects are inclusive of workplace stressors, dysfunctional leadership, poor social support, and work-related chronic stress caused by overcapacity that can contribute to, exacerbate, and incubate both short- and long-term health risks [8,9,10]. This gap in both research and practice is significant as long hours, job insecurity, and a lack of work-life balance influence poor health outcomes, including an increased risk for diabetes, early onset hypertension, and, in some cases, premature mortality [11].

Early indicators of change in health status are often used to predict risk for chronic health outcomes. The sympathetic nervous system is a network of nerves that activates the “flight or fight” response, triggered, in part, by catecholamines and resulting in accelerated heart rate, widened bronchial passages, constricted blood vessels, and increased blood pressure, among other outcomes. If overstimulated chronically, this otherwise beneficial physiological response can lead to detrimental health outcomes. Catecholamines can indicate sympathetic tone or stress by assessing the sympathetic nervous system response (i.e., norepinephrine, dopamine) [12] and the adrenal medullary secretion (i.e., epinephrine) [13], which have significant health implications for chronic diseases [14,15], including cardiovascular disease [16,17,18]. Increased levels of epinephrine are associated with high blood pressure and increased left ventricular mass [13,19,20] and both increased epinephrine and norepinephrine can cause platelet aggregation and secretion increasing overall cardiovascular disease risk [21]. Additionally, overactivation of catecholamines is consistently observed with chronic heart failure [22] and arrythmias, [23] possibly contributing to cardiac death. Catecholamines may contribute to or exacerbate other chronic health conditions, such as the development of diabetes [24] as well as the progression of diabetic neuropathy [15]; augmented catecholamine release and resistance are present in individuals with obesity [25,26,27]; as well as lead to an anxiety disorder [28]. The effects of chronic diseases negatively impact the US economy through factors such as lower rates of employment of individuals with chronic health conditions [29] and large annual expenditures for employee wellness programs. Despite billions invested in disease prevention and wellness by employers, US health spending approached $4.1 trillion, or $12,530 per person, in 2020, an increase of 9.7%, leading to financial strain for federal, state, and personal health systems [30].

Approximately 5–8% of annual healthcare costs are estimated to be associated with aspects of workplace culture and leadership [11,31], and these costs may be directly related to stress sequelae, including chronically increased catecholamines. Conversely, workplace cultures that engender positive, supportive communities also have a higher proportion of engaged employees that are more productive, creative, satisfied, profitable, and, perhaps in some cases, healthier. Much investigation of workplace culture focuses on employee engagement, retention, work satisfaction, and psychosocial-related outcomes. There is a gap in the literature on how workplace culture influences chronic diseases, such as heart disease or diabetes. These diseases often take a decade or more to manifest, making it hard to associate work conditions with future incidences of chronic disease in the absence of validated preclinical biomarkers of risk, such as catecholamines. Thus, objective health metrics for monitoring workplace culture could significantly impact the trajectory of workplace-related health outcomes. Objective quantitative measures are often preferred in health risk modeling as they can be directly linked to specific environmental factors such as stress. The current study addresses this gap in the literature by examining how specific aspects of workplace culture, including workplace community, employee engagement, workplace isolation, and the overall environment, impact physical health by associating subjective measures of work culture with objective preclinical biomarkers of health (i.e., catecholamines and their metabolites).

## 2. Methods

**Figure 1:**
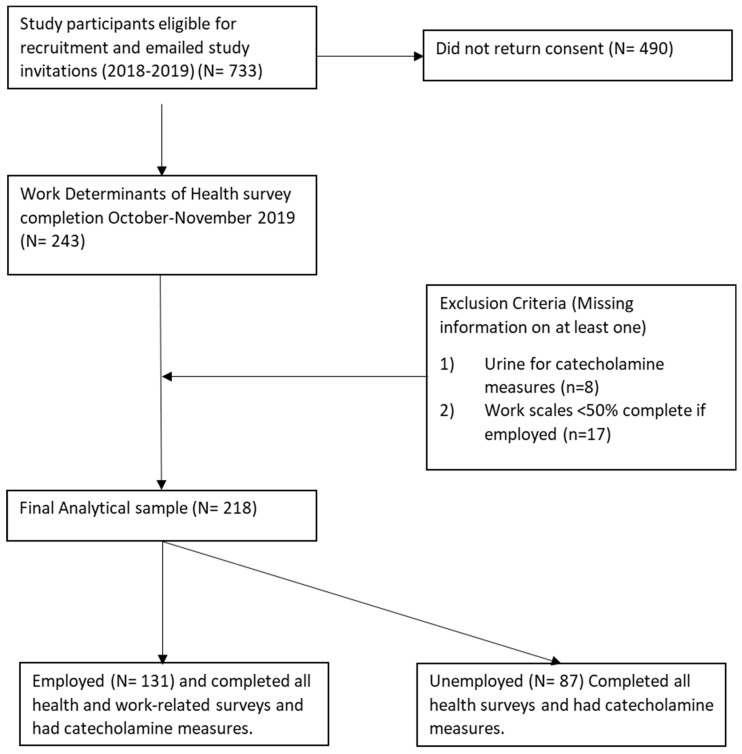
CONSORT Flow Diagram.

Participants from an ongoing cardiovascular risk cohort were recruited to participate in an online survey to investigate workplace culture and stress-related risk factors for chronic disease in South Louisville (Kentucky) during 2018–2019. All study-related procedures and measures were approved by the University of Louisville’s Institutional Review Board (IRB #15.126), and informed consent was obtained prior to administering questionnaires and collecting biological data. For this sub-study, 733 cohort participants received an emailed invitation to complete a series of 7 questionnaires that took approximately 25 min (IRB #19.1047). Of those emailed, 243 responded with a participation rate of 33%. Of those who participated, 17 were missing more than half of the questionnaire data and were excluded. Biologic samples were collected at study visits prior to the administration of sub-study questionnaires, and 8 urine samples were missing; thus, the analytic sample for the current study is 218 participants (Figure 1). Social, demographic, and occupational information was collected by self-reported questionnaires at the initial visit and again as part of the sub-study. Initial questionnaires were completed while participants waited at health stations and/or prior to checkout; sub-study questionnaires were completed online. Responses were collected and managed using Research Electronic Data Capture (REDCap) electronic data capture tools hosted at the University of Louisville [32,33].

### 2.1. Participants

All participants were enrolled in the Health, Environment and Action in Louisville (HEAL) study from May to October 2018 and 2019. HEAL invited participants aged 25–70 years to a health study visit at various community locations within a 4-mile target neighborhood. Recruitment included mailings, community events, and electronic outreach (e.g., social media). At each in-person study visit, which took approximately 2 h, participants completed a range of questionnaires that included demographic information, medical status, occupational information, and lifestyle choices, as well as a series of physical measures. Biospecimens were collected, including non-fasted clean catch urines. Urine was processed and stored at −80 °C until analyzed. If a participant in the HEAL study had opted into future research opportunities and identified email as their preferred method of contact, they were emailed a study invitation and consent in October of 2019. After providing consent, participants were asked to complete questionnaires about their stress levels, depression history, employment history, and employment views. Questionnaires were administered through REDCap and linked to previously collected health information. All questionnaires reported acceptable levels of standardized reliabilities, ranging from 0.83 to 0.94, indicating high levels of overall scale reliability. Compensation was provided for time.

### 2.2. Psychosocial Questionnaires

To assess levels of stress, participants responded to a perceived stress scale first proposed by Cohen et al. [34]. This measure, one of the most commonly used scales to examine perceptions of stress, is a 10-question 5-point Likert scale with responses ranging from never to very often (Appendix A). The questions are summed, with 4-positively stated items reverse scored to achieve a final score for the scale. The reported standardized Cronbach alpha for the perceived stress scale in the current study was 0.91.

To assess depression, the Patient Health Questionnaire-9 (PHQ-9) was administered [35]. The scale scores are the sum of 9 questions that are part of the DSM-IV criteria for depression, and responses range from 0 (not at all) to 3 (every day). Scores of 5, 10, 13, and 20 indicate mild, moderate, moderately severe, and severe depression, respectively (Appendix B).

To assess mental well-being, the Warwick-Edinburgh Mental Well-being Scale (WEMWBS) was administered to participants [36,37]. It covers the feeling and functioning domains of mental well-being over the past 2 weeks. This measure contains 14 positively worded items, each with a 5-point response scale from 1 (none of the time) to 5 (all of the time), and responses are summed for a total score (Appendix C). The reported standardized Cronbach alpha for the WEMWBS in the current study was 0.94.

To assess workplace isolation, the Workplace Isolation Scale-7 (WIS-7) was given to participants. This investigator-generated scale assessed the impact of feeling isolated and/or alone at work. The items covered feelings of being ignored, overlooked, and lonely at work or when working. Scoring for the WIS-7 was on a continuum from 1 (strongly disagree) to 5 (strongly agree). The higher the score, the more isolated and disconnected a participant was from their work colleagues. Higher scores could signal loneliness or feelings of being ignored at work. Each of the 7 items was averaged to create a sum score. The reported standardized Cronbach alpha for the WIS-7 in the current study was raw 0.953 and standardized 0.954.

To assess workplace community, the Workplace Community Scale-5 (WCS-5) was administered to those who reported current employment. This investigator-generated scale assessed the degree of feeling part of a community at work that is supported, connected, and promotes a sense of belonging. The questionnaire covers feelings of support, inclusion, and appreciation when at work or when working. Scoring for the WCS-5 was on a continuum from 1 (strongly disagree) to 5 (strongly agree). The higher the score, the higher the degree of belonging a participant experienced during and as a part of their working experience. Items were averaged to create a sum score. The reported standardized Cronbach alpha for the WCS-5 in the current study was 0.87.

The assess employee engagement, the Employee Engagement Scale (EES) was administered to participants. The scale has three subscale dimensions—cognitive engagement (e.g., I am really focused when I am working), emotional engagement (e.g., I feel a strong sense of belonging to my job), and behavioral engagement (e.g., I am willing to put in extra effort without being asked). Each subscale is comprised of two questions, for a total of 6 questions. All items were measured on a 5-point Likert scale, with 1 indicating strongly disagree and 5 indicating strongly agree. The psychometric properties of the EES have been described by Shuck et al. [38]. In previous research, the EES demonstrated strong internal consistency (*a* = 0.91) and acceptable model fit (CFI = 0.93; TLI = 0.91; c^2^_44_ = 741.17, *p* < 0.001). The reported standardized Cronbach alpha for the EES in the current study was 0.83.

To assess overall workplace culture, the Cognitive Workplace Appraisal Scale-11 (CWAS-11) was completed by participants who reported current employment. Each item on the CWAS-11 was designed to understand an antecedental dimension of workplace culture and was measured on a 5-point Likert scale, with 1 indicating strongly disagree and 5 indicating strongly agree. The psychometric properties of the CWAS-11 have been described previously [38]. In past research, the CWAS-11 demonstrated strong internal consistency (a = 0.87) and acceptable model fit (CFI = 0.99; TLI = 0.99; c^2^_51_ = 459.89, *p* < 0.001). The reported standardized Cronbach alpha for the CWAS-11 in the current study was 0.90.

### 2.3. Catecholamine Measures

Urinary levels of four parent catecholamines and six of their metabolites were measured by UPLC-MS/MS as described in previous work [39]. A list of all catecholamines and their metabolites can be found in Appendix D. Briefly, pre-stored (−80 °C) random catch urine samples were thawed on ice, and 5 µL of urine sample was mixed with 50 µL of deuterated internal standards (epinephrine-d_6_, norepinephrine-d_6_, dopamine-d_4_, metanephrine-d_3_, normetanephrine-d_3_, 4-hydroxy-3-methoxymandelic acid-d_3_, 4-hydroxy-3-methoxyphenyl-d_3_-acetic-d_2_ acid, 5-hydroxyindole-4,6,7-d_3_-3-acetic-d_2_ acid) and 195 μL of 0.2% formic acid in a 2 mL amber UPLC sample vial, and the samples were analyzed on a Xevo TQ-S micro quadrupole mass spectrometer with an ESI ionization source, interfaced with Waters Acquity Class-H UPLC equipped with a quaternary pump system (Waters, Milford, MA, USA). The analytes were resolved on an Acquity UPLC high strength silica (HSS) perfluorophenyl (PFP) (150 mm × 2.1 mm, 1.8 μm) column (Waters Inc., Milford, MA, USA) maintained at 40 °C, using a binary gradient consisting of 0.2% formic acid (Solvent A) and methanol (Solvent B). The gradient started with 0.5% solvent B at a flow rate of 0.4 mL/min for 1 min and then ramped up to 95% solvent B at a flow rate of 0.35 mL/min over a period of 3 min. The gradient was then maintained at these conditions for 0.5 min before recycling back to 0.5% solvent B in 0.1 min, and then held at 0.5% solvent B at a flow rate of 0.4 mL/min for 5.4 min. The MS/MS data were acquired in time-scheduled multiple reaction ion monitoring (MRM) mode using electrospray ionization. Polarity switching was used to detect positive and negative ions in the same run. The electrospray ionization inlet conditions were: capillary 0.50 kV, cone 28 V, source temperature 150 °C, desolvation temperature 600 °C, cone gas flow 50 L/h, and desolvation gas flow 1000 L/h.

### 2.4. Statistical Analysis

Summary statistics are included for the full data set. The categorical variables are summarized by frequency and percentages and continuous variables are summarized by mean (SD, standard deviation) and median (minimum, maximum). For comparing two or multi groups, the association with a categorical variable is based on a Chi-Square test (or Fisher’s Exact test if the expected cell count is less than 5). The analysis of variance (ANOVA) or Generalized Linear Model (GLM) procedure was used to compare continuous outcomes between two or multiple groups. Pearson’s correlation was used to measure the association between two continuous measures. For highly skewed variables log or logit transformation was used. Due to strong correlations between all catecholamines and catecholamine metabolites, we chose to use only 3 MT. This choice was related to a strong correlation with work culture surveys and previous literature supporting the importance of biological outcomes and some precedence of change due to stressors. As 3 MT is strongly correlated with other catecholamines and their metabolites, other catecholamines may behave similarly to 3 MT in relation to self-reported psychosocial work scales. Univariable logistic regression was used to evaluate the association between employment status and demographics and selected wellness scale variables and catecholamines; odds ratios and 95% confidence intervals (CIs) are reported. EES, WIS-7, and CWAS-11 were not included in the univariable logistic regression due to the response having zero variability. Multivariable linear regression was used to associate catecholamine level using selected wellness scale variables and selected demographics; regression coefficient and standard error are reported. Wellness scale variables and catecholamines were selected using Pearson’s correlation coefficients. Data were analyzed using SAS 9.4 (Cary, N.C.).

## 3. Results

The mean age of participants was 50 (±13) years, 66% reported being female, and 18% self-identified as a member of a minority population. Participants who reported current employment were more likely to be younger and report higher income and educational background (*p*-values < 0.001). Participants who reported they were not working scored higher on depression (6.20 ± 5.60 vs. 4.07 ± 4.74, *p* = 0.003) and stress scales (16.09 ± 8.58 vs. 13.41 ± 7.13, *p* = 0.013); conversely, participants who reported they were working scored higher on the well-being scale (52.88 ± 8.52 vs. 49.14 ± 9.78, *p* = 0.003). Some participants (n = 49) experienced employment changes: 28 participants reported working several months prior to completing these measures and 21 participants reported obtaining employment in the same timeframe (Table 1).

Levels of log transformed catecholamines and their metabolites were normalized to creatinine and compared between the individuals who reported current employment and those who do not. Higher mean levels of norepinephrine (NE [3.49 ± 0.05 vs. 3.34 ± 0.04, *p* = 0.008]), 5-hydroxytryptamine or serotonin (5 HT [4.33 ± 0.05 vs. 4.19 ± 0.03, *p* = 0.011]), normetanephrine (NMN [3.07 ± 0.04 vs. 2.96 ± 0.03, *p* = 0.36]), 3-methoxytyramine (3 MT [3.25 ± 0.04 vs. 3.12 ± 0.03, *p* = 0.007]) and 5-hydroxyindolacetic (5HIAA [7.95 ± 0.05 vs. 7.81 ± 0.04, *p* = 0.025]) were seen in those who did not currently hold a job compared with those who did. No other significance was seen for catecholamines or metabolites between the groups (Data not shown).

Bivariate correlations and summary statistics for health-related scales, including well-being, stress, and depression, were completed. Work-related questionnaires were completed by those currently employed to measure employee engagement, workplace culture, work isolation, and work community. Questionnaire data were skewed and thus logit transformed prior to correlations. All health-related and work-related surveys were significantly correlated, with well-being showing an inverse relationship to the other surveys (^#^ *p* ≤ 0.1; * *p* ≤ 0.05; ** *p* ≤ 0.01; *** *p* ≤ 0.001) as shown in Table 2. Importantly stress-related measures completed during initial in person visits and the repeated measure during this sub-study were highly correlated suggesting stability in the amount of stress across time. Similarly, all participants’ urinary catecholamines and catecholamine metabolites normalized to creatinine to account for urine concentration and log transformed were analyzed for bivariate correlations (Table 3). All catecholamines and catecholamine metabolites were significantly correlated (^#^ *p* ≤ 0.1; * *p* ≤ 0.05; ** *p* ≤ 0.01; *** *p* ≤ 0.001).

Bivariate analysis of health- and work-related scales and catecholamines are presented in Table 4. Higher depression scores were associated with higher levels of dopamine (DA [*r* = 0.21]), 5 HT (*r* = 0.19), 3 MT (*r* = 0.19), and metanephrine (MN [*r* = 0.15]), and inversely associated with epinephrine (EPI [*r* = −0.17]). Higher reported stress was associated with significant correlations between DA (r = 0.27), 5 HT (*r* = 0.18), and 3 MT (*r* = 0.26), and inversely associated with EPI (*r* = −0.21). Participants with higher reported well-being had lower levels of DA (*r* = −0.28), 5 HT (*r* = −0.17), and 3 MT (*r* = −0.31). Participants who reported experiencing a more engaged workplace had lower levels of DA (*r* = −0.20) and 3 MT (*r* = −0.22). Those who reported more positive workplace culture had lower levels of DA (*r* = −0.17) and 3 MT (*r* = −0.26) and higher levels of NE (*r* = 0.19) and 5 HIAA (*r* = 0.22). When participants identified more workplace isolation, they had higher levels of DA (*r* = 0.19) and 3 MT (*r* = 0.19). Positive workplace culture was correlated with lower levels of DA (*r* = −0.22) and 3 MT (*r* = −0.28) and higher levels of 5 HIAA (*r* = 0.22) and HVA (*r* = 0.20).

Given high levels of correlations between catecholamines and the metabolites, we chose 1 catecholamine metabolite, 3 MT, of dopamine for univariable analysis and multivariable analysis with a priori selected demographic variables that may confound work experience as well as creatinine (Table 4). In univariable linear regression, we saw significant positive associations for biological sex, race perceived stress (PSS1 and PSS2), and depression (PHQ-9). We saw significant negative associations for higher education, increased wages, higher employee engagement (EES), higher workplace culture (CWAS-11), and higher wellbeing (WEMBS1). (Table 5). As seen in Table 6, for multivariable regression of 3 MT, we modeled employment engagement, work culture, and workplace isolation in separate models to explore the relationship after adjusting for sex, race, income, and creatinine. In fully adjusted models, we saw that in environments with a more positive workplace culture, levels of 3 MT remained significantly lower (β = −0.065 ± 0.025, *p* = 0.01). This model accounted for 23% of the variance seen in 3 MT levels. In fully adjusted models, the relationship between increased workplace isolation and higher 3 MT remained significant (β = 0.064 ± 0.030, *p* = 0.04) and accounted for 22% of the variance seen with 3 MT levels. Employee engagement lost significance when fully adjusted (β = −0.056 ± 0.030, *p* = 0.06).

## 4. Discussion

If work culture can contribute to health disparities and/or influence health and health risk variability, a better understanding of these relationships is needed. Connecting workplace culture with specific catecholamine metabolites is a significant step in understanding how our work environment impacts our physical health. This study is one of the first to correlate objective and quantitative measures of biological data, such as urinary levels of catecholamines and their metabolites, with a questionnaire and self-reported social science measures of workplace culture. In this study, we found a strong positive correlation between the quantitative measures and subjective stress scales for dopamine and the primary metabolite 3 MT as well as a strong inverse correlation with epinephrine. The findings suggest that work conditions may be important regulators of the sympathetic tone and thereby significant modifiers of cardiovascular disease risk [12,40,41], mental health [42,43], and metabolic disorders such as diabetes [15,24] and obesity [26].

Another major concern to the workforce is stress. Most workers understand that long hours, persistent levels of stress caused by heavy workloads and poor leadership, as well as dysfunctional cultures impact their health. Effects of such experiences may include poor sleep quality, frustration, and mood swings, as well as high blood pressure [44]. Previous studies have shown that employees’ catecholamines increased with feelings of time or demand pressures [45]. Our findings suggest that work-related social environments can also have objectively measured impacts on stress markers as evidenced by the changes with 3 MT. 3 MT, a metabolite of dopamine has the potential for detrimental health outcomes as it is an important regulator of blood pressure [46,47]; can affect hormones or other agents that are important for cardiovascular health including aldosterone, angiotensin, ANP, insulin, and nitric oxide [47]; help regulate food intake [48]; has addiction implications [49]; is linked to aggressive activities [50]. Empirically, these findings highlight that not only does negative workplace culture have some immediate consequences, but also that chronically poor workplace culture could influence long-term health risks, contributing to sickness and absenteeism, inflating health insurance costs, lowering the overall quality of life, and impacting employee performance. Indeed, Payne et al. found workplace culture and more specifically reported leadership support, coworker support, and employee engagement were all associated with perceived support for health whereas perceptions of lifestyle risk were associated with the environment and policy in the workplace [51]. In short, a cascading set of consequences may exist for workplaces with negative organizational cultures and their employees.

Importantly, our data showed statistically significant bivariate correlations between employee engagement, workplace culture, workplace isolation, and several catecholamines (See Table 4), signifying that when the culture was positive, participants experienced lower levels of DA and 3 MT and when work was isolating and perceived as lonely, those levels were higher. The fact that both the parent catecholamine and the primary metabolite show significant correlations strengthens the findings. Due to the high degree of correlation between catecholamines, it is possible that other catecholamines will behave similarly to 3 MT in relation to self-reported psychosocial work scales. Previous research supports that work environments that increase catecholamines can concurrently increase blood pressure [52]. These data suggest that stress and sympathetic tone may be directly impacted by non-traditional workplace environments. It will be essential to continue to develop measures and metrics to guide interventions at the work culture level to mitigate future health risks and disparity.

Previous studies suggested a relationship between other aspects of the workplace environment and catecholamine changes [45,53,54,55,56], but none focused exclusively on the aspects of workplace culture assessed here. Interestingly, our bivariate analysis showed an increase in norepinephrine in those individuals who reported a more positive workplace. Norepinephrine is a neurohormone that is often associated with stress responses but is also associated with arousal, alertness, and attention can be stimulated by foods and drinks with caffeine [57], nicotine [58], and exercise [59] in addition to changing circadian rhythm [60]. This suggests that the function of norepinephrine is more complex than its simple association with the flight or fight response alone may suggest. The main effect of sympathetic activation is often dictated by which norepinephrine receptor is activated and can be associated with memory formation and consolidation [61], and switching between mental focus and flexibility [62]. Importantly, previous research suggests increases in epinephrine and norepinephrine lead to increases in performance and focus as well as social adjustment and emotional stability [63]. It is possible that the small increases we see in our study may be indicative of engagement in the workplace vs. a stress response. Alternatively, in individuals with some chronic conditions, such as ADHD, chronic fatigue syndrome [64], fibromyalgia [65], bipolar disorder [42], depression [43], and migraines [66], norepinephrine levels are lower and at times its release in response to stimuli is blunted.

Little research has examined work culture, engagement, and isolation in relation to quantitative markers of stress. This avenue of research may have been limited, in part, due to the complicated nature of measuring objective outcomes such as catecholamines. Research has shown that urinary and plasma epinephrine increased with acute stress, but there is considerable variance in sample variations suggesting sampling is time sensitive and for plasma catecholamines frequent sampling would be needed [67]. Additionally, it should be noted that both rewards and mild stress increase dopamine, with social defeat as the largest inducer of increased dopamine [68]. Current research suggests that mild acute stress increases dopamine, whereas chronic stress has the potential to reduce both baseline levels of released dopamine, and in particular the pre-frontal cortex [69]. The pre-frontal cortex has been implicated in executive function that includes planning, personality, decision making, and social behavior. More importantly, executive function helps to determine future consequences of current activities, working toward a defined goal, prediction of outcomes, and expectation-based action, all of which are important in the workplace.

Psychosocial stress is highly associated with hypertension and cardiovascular disease. In a multi-ethnic study, researchers found that participants with increased urinary levels of nor-epinephrine, epinephrine, and dopamine had an increased risk of hypertension, and this risk was higher for individuals who were <60 years of age [70]. Another study found that perceived job demands of working under time pressure or working behind schedule did not elicit self-reported health complaints [71], despite the potential to be a psychosocial stressor. Increased dopamine release has been linked to increased atrioventricular conduction [72], which can lead to rhythm changes in the heart. Decreased dopamine was associated with an increase in heart rate variability, a sign of heart health [72]. Not only did our findings show significant correlations, but, through modeling, workplace culture and isolation accounted for a significant portion of the explained variance in 3 MT. These observations support the notion that social experiences of work contribute to an employee’s overall health profile and their risk for long-term chronic disease, making the case for workplace culture to be considered within the broader domains of health and well-being. Taken together, these findings suggest the need for continued investigation of workplace culture and employee health. For example, when culture is positive, does long-term chronic risk go down for events such as heart disease, heart attacks, and early-onset hypertension, and will executive functioning improve?

Although myriad factors may influence an individual’s health, our findings indicate that at least some portion of an employee’s health could be influenced by social and psychological factors in workplace environments within the overall Work Determinants of Health™ model. Our findings underscore that models of health and wellness in the workplace might consider organizational culture as a component of their overall focus. Many wellness-type programs are driven by traditional disease risk modifiers that require active participation from the employee, necessitate the use of free time outside work, and may create additional burdens for the employee. Often, employees simply return to a work environment that exacerbates the health complications they experience, potentially promoting a vicious and dysfunctional cycle. In addition, often communities at work that are the most isolated are those who have been traditionally marginalized, making a clear case for health and diversity, equity, and inclusion initiatives. Perhaps a positive workplace culture is a health and wellness benefit as much as it is an experience. For decades, traditional assumptions regarding wellness in the workplace rested on the idea that it was an employee’s responsibility to be proactive in managing their health, often through various programs provided by their employer. In a related vein, our findings also have implications for the rising costs of health and healthcare. Using only the markers that we have identified in this study (i.e., DA and 3 MT), we can point to risks that burden the financial health of individual employees and the overall organization.

We believe our study design employing objective and self-reported data provides valuable insights about work and overall health. This study focused on the social workplace environment; we cannot discount the influence of other environmental factors on catecholamines. More comprehensive studies should be conducted to allow for control of additional factors known to change catecholamine excretion such as noise [73], fasting, low protein diets [74], and smoking [72]. Exercise has been shown to decrease the urinary excretion of dopamine and increase the urinary excretion of epinephrine [75]. Our study size was limited; we did not explore sex specific physiological stress responses, in particular, seen in the hypothalamic-pituitary-adrenal axis, responsible for the release of epinephrine and norepinephrine as well as the autonomic nervous system [76]. For this sub-study of a larger population-based study, questionnaire completion occurred outside routine study visits over a limited timeframe, resulting in a 33% response rate. Our urine samples were a clean catch, rather than 24 h ones; thus, we did not look at diurnal or intra-individual variations in catecholamines, however, 24-h urines may mask differences due to dilution of our outcome measures. We did adjust our data for dilution and, when possible, for confounding. Despite these adjustments, we were not able to eliminate the possibility of residual confounding, making it important for future studies to investigate causation and mechanisms linking work culture to health risk. Our multi-disciplinary approach embedded in a health study will allow us to collect valuable data on confounding in the future.

## 5. Conclusions

In summary, the data suggest a significant and important relationship between how we experience work and quantitative markers of our overall health. These findings could motivate organizations to consider including workplace culture as part of an overall wellness plan and in support of building cultures where employees flourish, feel connected, and are engaged. Additionally, longitudinal studies would be of benefit, to include multi-time wave analysis and intervention work within specific organizational settings.

## Figures and Tables

**Table 1:** Characteristics of study participants by employment status.

Variables	Total (N = 218)	Not Employed (N = 87)	Employed (N = 131)	*p* Value
Age (*Mean ± SD)*	50 ± 13	56 ± 12	46 ± 12	<0.001
Race (n, %)				1.000
White (%)	180 (83)	72 (83)	108 (82)	
Black (%)	30 (14)	12 (14)	18 (14)	
Other (%)	8 (4)	3 (3)	5 (4)	
Sex (n, %)				0.471
Female (%)	144 (66)	55 (63)	89 (68)	
Male (%)	74 (34)	32 (37)	42 (32)	
Household Income (n, %)				<0.001
less than $20,000 (%)	38 (17)	26 (30)	12 (9)	
$20,000–44,999 (%)	66 (30)	30 (34)	36 (27)	
$45,000–64,999 (%)	52 (24)	17 (20)	35 (27)	
greater than $65,000 (%)	55 (25)	12 (14)	43 (33)	
Education (n, %)				<0.001
Highschool diploma/GED or below (%)	54 (25)	33 (38)	21 (16)	
Some college to 4 year degree (%)	131 (60)	46 (53)	85 (65)	
Graduate degree (%)	32 (15)	8 (9)	24 (18)	
Missing	1	0	1	
Job status change (n, %)				<0.001
None (%)	138 (63)	27 (31)	111 (85)	
Changed (%)	80 (37)	60 (69)	20 (15)	
Health scales *(Mean ± SD)*				
PHQ-9	4.92 ± 5.19	6.20 ± 5.60	4.07 ± 4.74	0.003
PSS1	14.48 ± 7.83	16.09 ± 8.58	13.41 ± 7.13	0.013
PSS2	26.28 ± 8.28	27.18 ± 8.57	25.68 ± 8.05	0.192
WEMWBS1	51.39 ± 9.21	49.14 ± 9.78	52.88 ± 8.52	0.003
WEMWBS2	47.80 ± 10.63	46.38 ± 11.43	48.74 ± 10.00	0.108

Health scales were summed. PHQ-9 = Patient Health Questionnaire-9 (PHQ-9). PSS1 = perceived stress scale wave 1. PSS2 = perceived stress scale wave 2. WEMWBS1 = Warwick-Edinburgh Mental Well-being Scale wave 1. WEMWBS2 = Warwick-Edinburgh Mental Well-being Scale wave 2. Significance was set at *p* ≤ 0.05.

**Table 2:** Bivariate correlations and summary statistics for health and work-related surveys.

Pearson’s Correlation Coefficients									
Variable	1	2	3	4	5	6	7	8	9
1. PSSR	1								
2. PHQ-9	0.43 ***	1							
3. PSS1	0.60 ***	0.68 ***	1						
4. WEMWBS1	−0.51 ***	−0.61 ***	−0.73 ***	1					
5. WEMWBS2	−0.72 ***	−0.48 ***	−0.57 ***	0.74 ***	1				
6. EES	−0.24 **	−0.16 ^#^	−0.28 **	0.29 ***	0.28 **	1			
7. WCI-5	−0.46 ***	−0.17 ^#^	−0.36 ***	0.47 ***	0.53 ***	0.60 ***	1		
8. WIS-7	0.41 ***	0.26 **	0.26 **	−0.36 ***	−0.45 ***	−0.31 ***	−0.44 ***	1	
9. CWAS-11	−0.40 ***	−0.28 **	−0.35 ***	0.41 ***	0.48 ***	0.43 ***	0.72 ***	−0.56 ***	1

PSS2 = perceived stress scale wave 2. PHQ-9 = Patient Health Questionnaire-9 (PHQ-9). PSS1 = perceived stress scale wave 1. WEMWBS1 = Warwick-Edinburgh Mental Well-being Scale wave 1. WEMWBS2 = Warwick-Edinburgh Mental Well-being Scale wave 2. EES = The Employee Engagement Scale. WCI-5 = Work Community Index. WIS-7 = The Work Isolation Scale. CWAS-11 = The Cognitive Work Appraisal Scale-11. Significance was set at ^#^ *p* ≤ 0.1; * *p* ≤ 0.05; ** *p* ≤ 0.01; *** *p* ≤ 0.001.

**Table 3:** Bivariate correlations and summary statistics for normalized catecholamines and their metabolites.

Pearson’s Correlation Coefficients										
Variable	1	2	3	4	5	6	7	8	9	10
1. NE	1									
2. DA	0.42 ***	1								
3. 5 HT	0.35 ***	0.58 ***	1							
4. NMN	0.82 ***	0.41 ***	0.34 ***	1						
5. 3 MT	0.37 ***	0.74 ***	0.47 ***	0.55 ***	1					
6. MN	0.33 ***	0.27 **	0.18 *	0.41 ***	0.25 **	1				
7. EPI	0.34 ***	0.12	0.04	0.32 ***	0.13	0.62 ***	1			
8. 5 HIAA	0.34 ***	0.17 *	0.26 **	0.31 ***	0.07	0.28 **	0.12	1		
9. HVA	0.23 **	0.16 ^#^	0.16 ^#^	0.22 **	0.13	0.16 ^#^	0.15 ^#^	0.28 **	1	
10. VMA	0.69 ***	0.39 ***	0.35 ***	0.67 ***	0.30 ***	0.31 ***	0.19 *	0.40 ***	0.32 ***	1

Data were normalized ng/mg creatinine and log transformed. NE = norepinephrine, DA = dopamine, 5 HT = 5-hydroxytryptamine or serotonin, NMN = normetanephrine, 3 MT = 3-methoxytyramine, MN = metanephrine, EPI = epinephrine, 5 HIAA = 5-hydroxyindolacetic, HVA = homovanillic acid, VMA = vanillylmandelic acid. Significance was set at ^#^ *p* ≤ 0.1; * *p* ≤ 0.05; ** *p* ≤ 0.01; *** *p* ≤ 0.001.

**Table 4:** Bivariate correlations and summary statistics for health and work-related surveys and normalized catecholamines and their metabolites.

Pearson’s Correlation Coefficients										
Variable	Age	PSS2	PHQ-9	PSS1	WEMWBS1	WEMWBS2	EES	WCI-5	WIS-7	CWAS-11
NE	0.34 ***	−0.10	−0.02	−0.10	0.04	0.07	0.11	0.19 *	−0.03	0.10
DA	−0.18 *	0.27 **	0.21 *	0.27 **	−0.27 **	−0.28 **	−0.20 *	−0.17 ^#^	0.19 *	−0.22 *
5 HT	0.03	0.19 *	0.19 *	0.20 *	−0.23 **	−0.17 ^#^	0.02	0.05	0.10	−0.02
NMN	0.33 ***	−0.01	0.03	−0.04	0.01	0.02	0.10	0.09	0.01	0.05
3 MT	−0.04	0.25 **	0.19 *	0.22 *	−0.24 **	−0.31 ***	−0.22 *	−0.26 **	0.19 *	−0.28 **
MN	0.06	−0.10	−0.15 ^#^	−0.07	−0.04	0.05	−0.07	0.02	0.05	0.05
EPI	−0.01	−0.21 *	−0.17 *	−0.15 ^#^	0.04	0.11	0.06	0.11	−0.02	0.13
5 HIAA	0.34 ***	−0.02	−0.12	−0.07	0.10	0.08	0.11	0.22 *	0.01	0.22 *
HVA	−0.04	−0.06	−0.10	−0.23 **	0.10	0.05	0.03	0.13	−0.02	0.20 *
VMA	0.32 ***	−0.12	−0.07	−0.11	0.05	0.12	0.06	0.14	−0.05	0.13

PSS2 = perceived stress scale wave 2. PHQ-9 = Patient Health Questionnaire-9. PSS1 = perceived stress scale wave 1. WEMWBS1 = Warwick-Edinburgh Mental Well-being Scale wave 1. WEMWBS2 = Warwick-Edinburgh Mental Well-being Scale wave 2. EES = The Employee Engagement Scale. WCI-5 = Work Community Index. WIS-7 = The Work Isolation Scale. CWAS-11 = The Cognitive Work Appraisal Scale-11. Catecholamine and metabolite data are normalized ng/mg creatinine and log transformed. NE = norepinephrine, DA = dopamine, 5 HT = 5-hydroxytryptamine or serotonin, NMN = normetanephrine, 3 MT = 3-methoxytyramine, MN = metanephrine, EPI = epinephrine, 5 HIAA = 5-hydroxyindolacetic, HVA = homovanillic acid, VMA = vanillylmandelic acid. Significance was set at ^#^ *p* ≤ 0.1; * *p* ≤ 0.05; ** *p* ≤ 0.01; *** *p* ≤ 0.001.

**Table 5:** Univariable linear regression of demographics, psychosocial and work scales to predict 3 MT level.

Variable	Comparison	Estimate	Standard Error	*p* Value
Sex	Female vs. Male	0.213	0.050	<0.001
Race	Other vs. White	0.181	0.063	0.005
Log age		0.05	0.088	0.567
Education	College vs. Highschool	−0.159	0.058	0.006
	Graduate Degree vs. Highschool	−0.142	0.079	0.075
Income	≥$65,000 vs. <$20,000	−0.291	0.073	<0.001
	$20,000-$44,999 vs. <$20,000	−0.105	0.071	0.139
	$45,000-$64,999 vs. <$20,000	−0.24	0.074	0.001
op	Yes vs. No	0.083	0.050	0.102
Household number	Two vs. one	0.035	0.061	0.57
	Three vs. one	0.033	0.061	0.594
Creatinine		<0.001	<0.001	0.924
PSS2		0.085	0.031	0.007
PHQ-9		0.104	0.036	0.004
EES		−0.08	0.031	0.010
CWAS-11		−0.082	0.025	0.001
PSS1		0.09	0.033	0.008
WEMBS1		−0.086	0.028	0.003

Variables are log transformed and work scales are logit transformed. PSS2 = Perceived stress scale wave 2. PHQ-9 = Patient Health Questionnaire-9 EES = The Employee Engagement Scale. CWAS-11 = The Cognitive Work Appraisal Scale-11. WIS-7 = The Work Isolation Scale. WEMWBS1 = Warwick-Edinburgh Mental Well-being Scale wave 1. 3 MT = 3-methoxytyramine. Significance was set at *p* ≤ 0.05.

**Table 6:** Multivariable linear regression of work scales to predict 3 MT level.

Work Scale	Estimate	Standard Error	*p* Value	R^2^
EES	−0.057	0.030	0.063	0.215
CWAS-11	−0.065	0.025	0.010	0.234
WIS-7	0.064	0.030	0.036	0.221

Variables are log transformed and work scales are logit transformed. Models were adjusted for sex, race, income, and creatinine. EES = The Employee Engagement Scale. CWAS-11 = The Cognitive Work Appraisal Scale-11. WIS-7 = The Work Isolation Scale. 3 MT = 3-methoxytyramine. Significance was set at *p* ≤ 0.05.

## Data Availability

The data presented in this study are available on request from the corresponding author. The data are not publicly available due to privacy issues.

## Appendix A. Perceived Stress Scale [22]

For each questions choose from the following alternatives: 0 = never; 1 = almost never; 2 = sometimes; 3 = fairly often and 4 = very often.

1. In the last month, how often have you been upset because of something that happened unexpectedly?
2. In the last month, how often have you felt that you were unable to control the important things in your life?
3. In the last month, how often have you felt nervous and stressed?
4. In the last month, how often have you felt confident about your ability to handle your personal problems?
5. In the last month, how often have you felt that things were going your way?
6. In the last month, how often have you found that you could not cope with all the things that you had to do?
7. In the last month, how often have you been able to control irritations in your life?
8. In the last month, how often have you felt that you were on top of things?
9. In the last month, how often have you been angered because of things that happened that were outside of your control?
10. In the last month, how often have you felt difficulties were piling up so high that you could not overcome them?

## Appendix B. Patient Health Questionnaire-9 [23]

Using the following options 1 = not at all; 2 = several days; 3 = more than half the days and 4 = nearly every day please answer the following questions: Over the past 2 weeks how often have you had…….

1. Little interest or pleasure in doing things?
2. Feeling down, depressed, or hopeless?
3. Trouble falling or staying asleep, or sleeping too much?
4. Feeling tired or having little energy?
5. Poor appetite or overeating?
6. Feeling bad about yourself—or that you are a failure or have let yourself or your family down?
7. Trouble concentrating on things, such as reading the newspaper or watching television?
8. Moving or speaking so slowly that other people could have noticed? Or so fidgety or restless that you have been moving a lot more than usual?
9. Thoughts that you would be better off dead, or thoughts of hurting yourself in some way?

## Appendix C. Warwick-Edinburgh Mental Well-Being Scale [25]

Please use the following options to respond how you have been feeling over the past 2 weeks, 1 = None of the time; 2 = Rarely; 3 = Some of the time; 4 = Often and 5 = All of the time.

1. I’ve been feeling optimistic about the future
2. I’ve been feeling useful
3. I’ve been feeling relaxed
4. I’ve been feeling interested in other people
5. I’ve had energy to spare
6. I’ve been dealing with problems well
7. I’ve been thinking clearly
8. I’ve been feeling good about myself
9. I’ve been feeling close to other people
10. I’ve been feeling confident
11. I’ve been able to make up my own mind about things
12. I’ve been feeling loved
13. I’ve been interested in new things
14. I’ve been feeling cheerful

## Appendix D. Free Biogenic Monoamine and Their Metabolites

Analyte	Abbreviation
Epinephrine	EPI
Norepinephrine	NE
Dopamine	DA
Serotonin	5-HT
Metanephrine	MN
Normetanephrine	NMN
3- methoxytyramine	3-MT
Homovanillic acid	HVA
Vanillylmandelic acid	VMA
5-hydroxyindole-3-acetic acid	5-HIAA
